# Should I stay for local hormone therapy or should I go for radiofrequency to treat vulvovaginal atrophy? A patient preference trial

**DOI:** 10.1097/GME.0000000000002393

**Published:** 2024-07-01

**Authors:** Chiara MF Dell'Utri, Elisabetta Manzoni, Irene Bonfanti, Francesca Marrocco, Giussy Barbara, Paola Pifarotti, Francesca Chiaffarino

**Affiliations:** From the 1 Gynecology Unit, Fondazione IRCCS Ca' Granda Ospedale Maggiore Policlinico, Milan, Italy; 2 Department of Clinical Sciences and Community Health, Università Degli Studi di Milano, Milan, Italy; 3 Obstetric and Gynecological Emergency Department and Service for Sexual and Domestic Violence, SVSeD, Fondazione IRCCS Ca' Granda Ospedale Maggiore Policlinico, Milan, Italy.

**Keywords:** Dynamic quadripolar radiofrequency, Energy-based device, Genitourinary syndrome of menopause, Patient preference, Topical estrogen therapy, Vulvovaginal atrophy

## Abstract

**Objective:**

To compare patient satisfaction rate in postmenopausal women who chose dynamic quadripolar radiofrequency or topical estrogens as their preferred treatment for genitourinary syndrome of menopause.

**Methods:**

Patients were divided into two groups according to their preference: one was treated with estrogen therapy (ET) and the other with dynamic quadripolar radiofrequency treatment (RF). All patients included fulfilled a series of validated questionnaires, at baseline and at the 6-mo follow-up, in order to evaluate the discomfort degree associated with the presence of vulvovaginal atrophy and the impact of the reported symptoms on QoL and sexuality.

**Results:**

After propensity score matching, the proportion of women considering themselves satisfied with their genital health conditions was extremely small at study entry (5.2% of the RF group and 6.9% of the ET group), while at a 6-mo follow-up, it increased to 46.7% and 46.6%, respectively. No statistically significant between-group differences were found regarding mean numerical rating scale scores for dryness and dyspareunia at follow-up (5.6 ± 2.6 vs 5.3 ± 2.3, *P* = 0.5; and 2.9 ± 2.5 vs 3.0 ± 2.7, *P* = 0.46). At 6-mo follow-up, we observed no statistically significant differences between the two groups regarding the other items evaluated. RF treatment was overall well tolerated.

**Conclusion:**

The use of quadripolar radiofrequency devices seems effective, but it is not associated with better clinical outcomes compared with topical hormone treatment, which is a substantially cheaper and more convenient treatment for genitourinary syndrome of menopause. Therefore, we suggest limiting the use of dynamic quadripolar radiofrequency selectively when topical estrogens are not effective, not tolerated, or contraindicated.

## Abstract 2

The use of quadripolar radiofrequency devices seems effective, but it is not associated with better clinical outcomes compared with topical hormone treatment.

The population of developed countries is aging considerably due to an increasing life expectancy.^1^ From this perspective, improving the quality of life (QoL) of the older population has become essential, and this also entails ensuring these individuals an adequate sexual life.^2^

In the last decades, there has been an increasing interest in sexual health, and a special consideration has been given to menopause; although society tends to consider postmenopausal women less interested in their sexuality, many women continue to consider sex as a fundamental element of their lives.^3^ Women are now more careful and demanding about their body, their sexual function, and their sexual image.

It is acknowledged that the hormone withdrawal that occurs following menopause may worsen sexual function for women by reducing sexual desire and lubrification and by causing dyspareunia.^4^ In fact, hypoestrogenism causes loss of collagen fibers and adipocytes in tissues, reduced vascular flow, reduced vaginal secretion and lubrication, and a rise in vaginal pH.^5^ It is estimated that almost 40% to 60% of all postmenopausal women suffer from genitourinary syndrome of menopause (GSM), although the actual number is likely underreported.^6^ GSM was described by The Menopause Society (formerly known as The North American Menopause Society) in 2014 as a clinical condition, which is due to the postmenopausal reduction in the concentration of sexual hormones and is characterized by vulvovaginal (dryness, burning, itching, vaginal discharge, bleeding, or spotting) and lower urinary tract symptoms (dysuria, urinary frequency, or recurrent urinary tract infections).^7^ This is a very common chronic and progressive condition that negatively interferes with female postmenopausal quality of life (QoL), with a significant negative impact on physical, emotional, and sexual life.

In this perspective, several therapeutic strategies have been proposed for the treatment of GSM. The Menopause Society suggests the use of low-dose local estrogen therapy as a first-line treatment for women with GSM.^8^ Local estrogens are highly effective in reverting physiological changes associated with GSM. They foster vaginal cell growth and maturation, increasing vaginal epithelium thickness and elasticity, favor lactobacillus recolonization, lower the vaginal pH, improve blood flow, and consequently enhance sexual function. Adverse effects of vaginal estrogen therapy are uncommon.^9^

However, the fear that some women may have of hormone therapy may lead to a reduction in its acceptance, in adherence, and consequently in the overall efficacy of the treatment. Other reasons for poor compliance include the inconvenience of the application of such treatment and vaginal discharge.^10^

During the last decades, however, a growing body of evidence has reported interesting but conflicting results regarding the use of energy-based devices as an alternative treatment for GSM described as even more effective than topical estrogen.

It has been suggested that the application of laser energy (fractional CO_2_, nonablative photothermal erbium:YAG) or of electromagnetic waves on the vaginal mucosa induces reepithelialization, neoangiogenesis, and production of collagen fibers. Hence, these types of treatment are supposed to remodel the vaginal tissue from an atrophic postmenopausal state to a thickened, glycogen-rich, and well-vascularized lining.^11^

Among the alternative nonhormone treatments, laser (CO_2_ fractional laser and erbium laser) was the first to be studied and continues to be one of the most used methods. However, despite a great initial enthusiasm for this technique, now, the available literature is insufficient to draw definitive conclusions on its efficacy. Laser technology continues to be highly controversial, as there is no consistency in the existing evidence and the reality of the so-called vaginal rejuvenation is far different from what had been immagined.^12^ Some authors provocatively wonder if laser therapies are more like “the emperor's new clothes” rather than a meaningful advance in the treatment of GSM.^13^ In a recent randomized controlled trial (RCT) comparing laser versus sham treatment, at a 12-mo follow-up, no differences between the groups were observed as regards symptom severity, quality of life, vaginal health index, and histological findings. To date, two other RCTs comparing laser versus sham treatment have obtained similar results.^14,15^ In the first study, the authors concluded that the treatment response 12 wk after laser application was comparable between sham and laser treatment.^15^ In the second study, the primary outcome was a two-stage improvement in dyspareunia from baseline to 6 mo; sexual function was improved in both groups, suggesting a possible placebo effect.^14^ Moreover, the efficacy of carbon dioxide laser treatment compared to topical estrogen treatment in GSM is reviewed in a recent meta-analysis of six RCTs. The author found no difference between the two groups comparing the severity of symptoms, Female Sexual Function Index (FSFI) and quality of life questionnaires.^16^ On the other hand, several observational studies have shown how laser can be beneficial on postmenopausal women's quality of life, sexual arousal, and satisfaction.^17-22^ As a matter of fact, most of the available studies are limited by design, short-term follow-up, and small series of patients; lack a control group; and lack a comparison with standard treatments, and most studies are industry sponsored. Due to these limitations, the level of evidence for the use of laser is low and does not allow for definitive recommendations for its use in routine clinical practice.^13^ The Royal College of Obstetricians and Gynecologists (RCOG) suggests that laser therapy may have a role for the treatment of GSM but at present should only be used as part of clinical trials and is not recommended for routine treatment.^23^

A more recent and substantially less costly category of energy-based devices is represented by radiofrequency treatment (RF), which is the focus of our research. RF devices emit focused electromagnetic waves that heat underlying tissues without targeting melanin. The release of thermal energy appears to induce collagen contraction, neocollagenesis, and neovascularization, thus restoring the elasticity and moisture of the vaginal mucosa.^24^ These changes are clinically correlated with an improvement in urinary incontinence tests and with significantly higher scores on validated vaginal health scales.^25^

The penetrative capacity within the tissues of RF is typically deeper than that of laser as it uses a lower frequency and longer wavelengths. RF penetration depth is also dependent on modality; monopolar systems penetrate most deeply. Furthermore, RF systems with bipolar and multipolar configurations can deliver energy externally to the vulva as well as to the vaginal mucosal epithelium and lamina propria.^26^ Physical devices based on dynamic quadripolar radiofrequency technology emit focused electromagnetic waves generating heat upon meeting tissue impedance; temperatures between 40°C and 45°C would seem to induce collagen production through fibroblasts via the activation of heat shock proteins and initiation of the inflammatory cascade.^27^ Moreover, it seems that RF treatment may induce an increase of lactobacillus and a decrease in vaginal pH, and this condition could be beneficial for vaginal atrophy, in addition to collagen production.^28^

Among radiofrequency devices, the most recent is the low-energy dynamic quadripolar radiofrequency (DQRF) device, which is what we used in our research. Developed by the Italian company Novavision Group SpA (Misinto, Monza-Brianza, Italy), innovative DQRF technology is at the core of Novavision's EVA device used in our study. DQRF allows the operator to define the depth and volume of the target area and reduce the quantity of administered energy. The DQRF device also allows electronic control of movements and temperature sensors to convey energy with high tridimensional precision using four steel dynamic electrodes.

Despite that many studies have described the use of energy-based devices, and especially radiofrequency devices, as successful and safe for GSM treatment, there is a lack of consensus on their use mainly because of a shortage of well-designed studies.^12,29-33^ In addition, in July 2018, the Food and Drug Administration (FDA) recommended caution in the use of all energy-based devices until regulatory approval is achieved. Hence, more robust data are needed to support the safety and efficacy of these devices.^34^

Given this background, we specifically aimed at investigating the effects of dynamic quadripolar RF compared with low-dose topical vaginal estrogens (estriol) on the reduction of GSM-related symptoms in postmenopausal women. To the best of our knowledge, currently, this is the first trial that exclusively focuses on this comparison.

Our primary endpoint was patient satisfaction with the proposed treatment at a 6-mo follow-up. Secondary endpoints included treatment effectiveness on dyspareunia and dryness, sexual function, and psychological well-being.

## METHODS

This is a patient preference, prospective parallel cohort study, aimed at comparing two different therapeutic options to treat GSM-related symptoms (dyspareunia and vulvovaginal dryness) in postmenopausal women: (1) topical vaginal estrogens; (2) nonsurgical radiofrequency thermal therapy.

Eligible for this study were all Italian-speaking postmenopausal women who were referred to the Urogynecology and Menopause Unit, Department of Women's and Children's Health, Fondazione IRCCS Ca' Granda Ospedale Maggiore Policlinico, Milan, Italy, between January 2020 and June 2022 complaining about distressing symptoms related to GSM. We included all the patients who sought treatment for genitourinary syndrome-related symptoms, regardless of their satisfaction level expressed in the questionnaire.

A negative Pap smear was requested prior to the enrollment. Exclusion criteria were current vulvovaginal diseases (including benign, premalignant, and malignant HPV-related diseases, vaginal carcinoma, cervical cancer, lichen sclerosus, lichen planus); history of vaginal/pelvic radiation; connective tissue diseases (such as scleroderma); reconstructive pelvic surgery within the past 6 mo or previous pelvic surgery with the interposition of synthetic mesh (except for synthetic midurethral slings, unless they were exposed or extruded); being a carrier of pacemaker and/or electrical systems; having contraindications to the use of topical estrogens; being a current user of systemic or topical hormone therapy or having used such treatment 3 mo prior to recruitment; and having used local moisturizers or lubricants 2 wk prior to enrollment.

Patients who accepted to take part in the study were given detailed information regarding the two types of treatment in order to make an informed choice before being assigned to either group.^35^ We informed the patients about the two treatment modalities in terms of effectiveness based on current knowledge, possible side effects, and commitment required, stressing the need to go to the hospital for treatment sessions for the RF group and to adhere to the treatment regimen required for the ET group.

Women who chose the energy-based intervention (RF group) received four dynamic quadripolar radiofrequency treatments 14 ± 2 d apart from each other. Further maintenance RF session was carried out 6 mo after the last one (EVA, technology patented by Novavision Group SpA). This instrument produces focused electromagnetic waves with a maximum emission power of 55 W and is capable of generating radiofrequency fields with high regional precision on the underlying layers.^36^ The device is also equipped with a specific sensor for detecting temperature and movements, designed to guarantee high safety (RSS radio frequency safety system). Each RF treatment lasted 15 min. The RF sessions were carried out by two female physicians. With the woman lying in a lithotomy position, the device probe was inserted into the vaginal canal. Throughout the treatment, the probe was pulled back and forth posteriorly to the hymenal ring with a continuous circular movement. Power was increased during the treatment, ranging from 24% to 32%. Probe temperature ranged from 39°C to 43.7°C. Any referred discomfort or side effect during the treatment was recorded.

Patients who chose local estrogen therapy (ET group) were instructed to use estrogen vaginal suppositories (estriol 0.5 mg) daily for the first 2 wk of treatment, following which the treatment was to be applied twice a week for 6 wk.

At study entry, all women underwent a thorough clinical evaluation and were asked to fill out a set of validated questionnaires aimed at evaluating GSM-related symptoms (dyspareunia and dryness) and the impact such symptoms had on their QoL and sexual function. At time of follow-up, women were asked to fill out the same questionnaires and extra questionnaires, evaluating satisfaction with treatment and individuals' impressions of change.

Dyspareunia and dryness were evaluated using a 0 to 10 numerical rating scale (NRS). Scores from 1 to 4 were considered indicative of mild symptoms, scores of 5 and 6 corresponded to moderate symptoms, and scores from 7 to 10 indicated severe symptoms.^37^

The Patient Global Impression of Severity (PGI-S) questionnaire was also used to evaluate symptom severity on a four-level scale: normal, mild, moderate, or severe.^38^

The Italian version of the Hospital Anxiety and Depression Scale (HADS) was used to evaluate women's psychological well-being. This questionnaire is composed of two seven-item scales, one for anxiety and one for depression. Each scale can be analyzed independently, although a total score can be calculated. The score ranges from 0 to 21, with higher scores indicating poorer psychological function.^39^

The Italian version of the FSFI was used to assess women's sexual function. The FSFI is a 19-item validated self-report questionnaire that assesses female sexual function with a specific focus on six different sexual domains: desire, arousal, lubrication, orgasm, satisfaction, and pain. Each subscale can be scored as an independent scale, although a full-scale score can be calculated. The overall score may vary between 2 and 36, with scores equal to or lower than 26.5 being suggestive of sexual dysfunction.^40,41^

Patients' satisfaction about their genital clinical condition was evaluated using a five-level Likert scale (very satisfied, satisfied, neither satisfied nor dissatisfied, dissatisfied, very dissatisfied). Women who stated being “very satisfied” or “satisfied” were considered as satisfied, while those who reported being “neutral,” “dissatisfied,” or “very dissatisfied” were considered as dissatisfied. Women were also asked to fill out the Patient Global Impression of Change (PGI-C) questionnaire.^42^

The Patient Global Impression of Change Scale (PGI-C) was also used at time of follow-up. This is a 7-point Likert scale (7 = very much improved, 6 = much improved, 5 = minimally improved, 4 = no change, 3 = minimally worse, 2 = much worse, 1 = very much worse) that evaluates patients' perception of their global improvement following a given treatment.^43^ As recommended in the Consensus Statement by Dworkin et al, we analyzed the percentages of patients endorsing each of the seven response options in each treatment group separately.^44^ In addition, all participants were asked to report the overall tolerability of their treatment on a five-level Likert scale (very well tolerated, well tolerated, moderately tolerated, poorly tolerated, not tolerated).

Sociodemographic data and medical history were obtained for each patient from clinical interviews and medical records.

A Local Institutional Review Board (Comitato Etico Milano Area 2) approval was obtained, and all women signed an informed consent (protocol n. 599/2020bis, September 2, 2020).

### Statistical analysis

The propensity score method was used to adjust for baseline differences and facilitate the comparability between the two groups. Propensity scores were generated with age, age at menopause, body mass index (BMI), parity, smoking status, and previous hormone therapy use. The model was based on logistic regression by using the single nearest neighbor matching method. After propensity score generation, patients underwent a 1:1 nearest available matching of the logit of the propensity score with a caliper width of 0.20 of the standard deviation of the score.^45^ Categorical variables were described as frequencies and percentages and were compared using the Pearson's chi-square or Fisher's exact test, as appropriate. Continuous variables were described as means and standard deviations (SD).

Assuming an alpha level of 0.05 and a beta level of 0.8, a total sample size of 116 patients (58 in each group) would enable the detection of a statistically significant difference in therapy satisfaction between groups, given a percentage difference of 25%.^46^

The odds ratio and the corresponding confidence interval for prevalence of satisfaction at month 6 were obtained from a conditional logistic regression model.

All the analyses were performed using SAS software, version 9.4 (SAS Institute, Inc, Cary, NC).

## RESULTS

A total of 186 postmenopausal women were enrolled in the study and included in the analysis: 89 women (48%) preferred RF treatment and 97 (52%) the local estrogen therapy. At study entry, participants' mean age was 58.0 ± 6.5 y in the RF group and 57.8 ± 4 y in the ET group (*P* = 0.73). Mean age at time of menopause was 48.5 ± 6.4 y in the RF group and 50.3 ± 3.4 y in the ET group (*P* = 0.50). No statistically significant differences were observed between the two groups for any other demographic characteristics (see Supplemental Table S1, http://links.lww.com/MENO/B273).

The propensity score method was used to adjust for baseline differences between groups. After propensity score matching, 58 women for each group were selected to be compared. All women completed the follow-up study period. Table 1 reports the sociodemographic characteristics of the participants selected after the propensity score matching. The mean age was 57.4 ± 6.6 y in the RF group and 57.8 ± 4 y in the ET group, and age at menopause onset was 49.4 ± 5.2 y and 50.2 ± 3.3 y, respectively. After propensity score matching, no statistically between-group differences were found with regard to the type of menopause (spontaneous or iatrogenic), previous use of hormone, parity, and tobacco use. The mean BMI was 23.7 ± 4.2 for the RF group and 23.5 ± 3.1 for the ET group (*P* = 0.82) (Table 1).

**TABLE 1:** Demographic characteristics of the women included in the study after propensity score matching

Baseline demographic characteristics	RF group (N = 58)	Local estrogen group (N = 58)	*P*
Age (mean ± SD)	57.4 ± 6.6	57.8 ± 4	0.73
Age at time of menopause (mean ± SD)	49.4 ± 5.2	50.2 ± 3.3	0.99
BMI, N (%)			0.73
Underweight (<18)	6 (10.3)	4 (6.9)	
Healthy weight (18.0 to 24.9)	32 (55.2)	36 (62.1)	
Overweight (25.0 to 29.9)	16 (27.6)	16 (27.6)	
Obese (>30)	4 (6.9)	2 (3.5)	
Tobacco use, N (%)			0.23
No	50 (86.2)	45 (77.6)	
Yes	8 (13.8)	13 (22.4)	
Number of deliveries, N (%)			0.72
0	20 (34.5)	16 (27.6)	
1	22 (37.9)	24 (41.4)	
≥2	16 (27.6)	18 (31.0)	
Menopause, N (%)			1.00
Spontaneous menopause	53 (91.4)	53 (91.4)	
Iatrogenic menopause	5 (8.6)	5 (8.6)	
Previous hormone therapy, N (%)			0.80
Never	42 (72.4)	44 (75.9)	
Systemic	6 (10.3)	4 (6.9)	
Local	10 (17.2)	10 (17.2)	

BMI, body mass index; SD, standard deviation.

At study entry, a great majority of women were dissatisfied with their genital health conditions: only 5.2% of women in the RF group and 6.9% of women in the ET group reported being satisfied with their genital health condition. These women were equally included in the study because we recruited patients who complain about distressing symptoms related to GSM and who wanted treatment for these symptoms, including those who despite the symptoms still managed to be satisfied with their sexual life.

At a 6-mo follow-up, 46.7% of women in the RF group and 46.6% in the ET group considered themselves as “satisfied” with their genital health condition (*P* = 1). Therefore, a borderline statistically significant difference was found in the number of patients satisfied with the treatment, both at baseline and at the follow-up evaluation (Table 2).

**TABLE 2:** Number of patients considered themselves as “satisfied” about their genital health and satisfaction with the received treatment after propensity score matching

At 6-mo follow-up	RF group (N = 58)	Local estrogen group (N = 58)	*P*
	n (%)	n (%)	
Satisfaction with their genital health	27 (46.7)	27 (46.6)	1.00
Satisfaction with their treatment	37 (63.8)	26 (45.6)	0.05

RF, dynamic quadripolar radiofrequency treatment.

At a 6-mo follow-up, 63.8% of women in the RF group and 45.6% of women in the ET group considered themselves as “satisfied” about their treatment (*P* = 0.05) (Table 2). The odds ratio of therapy satisfaction at month 6 was 1.85 (95% confidence interval, 1.0 to 3.6), according to the conditional model.

At baseline, women enrolled in the two groups complained of a severe grade of dryness and dyspareunia (Table 3). Following treatment, an improvement was observed in both groups for both dryness and dyspareunia, with an increase of +2.6 (±2.5) points in the mean NRS score reported for dryness in both groups and an increase of +2.9 (±2.5) and +3.0 (±2.7) in the RF group and ET group, respectively. Mean NRS score for dryness and dyspareunia were lower at follow-up with no statistically significant differences between the two groups (Table 3).

**TABLE 3:** NRS for dryness and dyspareunia at baseline and follow-up by study group after propensity score matching

	Therapy		*P* (Wilcoxon test)
	RF group (N = 58)	Local estrogen group (N = 58)	
	Mean ± SD	Mean ± SD	
NRS dryness			
At baseline	8.2 ± 1.5	7.9 ± 1.0	0.08
At 6-mo follow-up	5.6 ± 2.6	5.3 ± 2.3	0.50
(Baseline − follow-up) delta	2.6 ± 2.5	2.6 ± 2.5	0.83
NRS dyspareunia			
At baseline	8.7 ± 1.4	8.3 ± 1.1	0.05
At 6-mo follow-up	5.8 ± 2.8	5.3 ± 2.3	0.19
(Baseline − follow-up) delta	2.9 ± 2.5	3.0 ± 2.7	0.46

NRS, numerical rating scale; RF, dynamic quadripolar radiofrequency treatment.

Mental health variables, measured with the HADS score, are shown in Table 4. Anxiety levels were similar in the two groups both at baseline and follow-up (7.2 ± 4.2 vs 6.1 ± 3.2, *P* = 0.23, and 6.1 ± 3.3 vs 5.9 ± 3.9, *P* = 0.70), while depression scores were greater in the RF group at baseline (6.9 ± 3.7 vs 5.2 ± 2.6, *P* = 0.01) but resulted equivalent at follow-up (5.3 ± 2.9 vs 4.8 ± 3.3, *P* = 0.38).

**TABLE 4:** HADS anxiety and depression at baseline and follow up after propensity score matching

	Therapy		*P* (Wilcoxon test)
	RF group (N = 58)	Local estrogen group (N = 58)	
	Mean ± SD	Mean ± SD	
HADS anxiety			
At baseline	7.2 ± 4.2	6.1 ± 3.2	0.23
At 6-mo follow-up	6.1 ± 3.3	5.9 ± 3.9	0.70
(Baseline – follow-up) delta	1.1 ± 4.0	0.3 ± 3.9	0.24
HADS depression			
At baseline	6.9 ± 3.7	5.2 ± 2.6	0.01
At 6-mo follow-up	5.3 ± 2.9	4.8 ± 3.3	0.38
(Baseline − follow-up) delta	1.6 ± 4.0	0.5 ± 2.9	0.10

HADS, Hospital Anxiety And Depression Scale; RF, dynamic quadripolar radiofrequency treatment.

Results regarding sexual function, measured with FSFI, are shown in Table 5. Following treatment, we observed an overall improvement in sexual function in both groups with an increase of 4.2 (±7.9) points for the RF group and 3.2 (±7.6) points for the ET group regarding the FSFI total score at follow-up versus baseline (Table 5).

**TABLE 5:** FSFI, total score, for baseline and follow up after propensity score matching

FSFI total score	Therapy		*P*
	RF group (N = 58)	Local estrogen group (N = 58)	
	Mean ± SD	Mean ± SD	
Baseline	11.6 ± 7.0	12.8 ± 6.7	0.34
Follow-up	15.8 ± 8.7	16.0 ± 7.6	0.93
(Baseline − follow-up) delta	4.2 ± 7.9	3.2 ± 7.6	0.59

FSFI, Female Sexual Function Index; RF, dynamic quadripolar radiofrequency treatment.

Disease severity (PGI-S) and patients' global impression of change (PGI-C questionnaire) are shown in Supplemental Table S2 (http://links.lww.com/MENO/B273). The majority of women in both groups, up to 50% of women in the RF group and 39.7% of women in the ET group, considered themselves as “very much improved or much improved,” and 29.3% of women in the RF group and 32.8% of women in the ET group considered themselves as “minimally improved.” At follow-up, 20.7% of participants in the RF group and 27.6% in the ET group considered themselves as not changed or even worsened.

Overall, RF treatment was well tolerated. We did not observe any adverse effects apart from one woman who reported at the beginning of one session a feeling of “electrical” discomfort in the vagina. In this case, the treatment was promptly interrupted, and the patient experienced an immediate spontaneous remission of the symptom. No anatomical lesions were observed at the inspection of the perineum and of the vagina.

## DISCUSSION

Our results show that RF and estrogen have a comparable effect on symptoms, satisfaction, and psychological well-being. As regards our primary outcome, at a 6-mo follow-up, we observed a comparable percentage of satisfied women in the two groups: 54.4% in the RT group and 42.4% in the ET group, respectively. Symptoms reduction and improvement in sexual function were also similar when comparing the two groups at time of follow-up.

The present study supports the hypothesis that RF devices relieve symptoms of GSM, although their efficacy appears comparable to that of topical estrogens, which are currently considered the first-line treatment.

Current FDA-approved treatments include moisturizers and lubricants, topical hormone therapy with estrogen or dehydroepiandrosterone (DHEA 0.50%, 6.5 mg), and oral selective estrogen receptor modulator (ospemifene 60 mg/d).^8^ Topical estrogens have been the gold standard treatment for vulvovaginal atrophy for years.^47^ However, this type of treatment entails disadvantages such as its inconvenience and leakage, which may determine suboptimal adherence. Percentage of discontinuation is reported to be greater than 86% for vaginal creams and greater than 50% for vaginal tablet.^48^ The length of our follow-up was only 6 mo; thus, we did not obtain data on long-term compliance. Moreover, in addition to the discomfort and not always optimal compliance of patients with local ET described in the literature, in some specific cases, hormone treatment may not be recommended (for instance, in the case of breast cancer survivors).

Among the alternative nonhormone treatments, radiofrequency devices are the most recent and, among these, the DQRF device is the latest arrival and the device used in our research.

Evidence on the use of RF is promising but for the moment is scarce and given only by observational studies especially for DQRF.^26^ Our results are consistent with other data obtained from observational studies that showed an improvement of GSM after RF treatment in postmenopausal women.^36,49-52^

Regarding the DQRF device, which is the focus of this research, in 2014, Nicoletti et al analyzed the effect of DQRF in an ex vivo/in vivo human experimental model: after a course of DQRF, native collagen underwent an immediate heat-induced rearrangement, and this lead to a spatial rearrangement and thickening of the collagen and elastic fibers, which displayed a skin reticular pattern, which is characteristic of young tissues.^36^ Benincà et al validated a four-session “EVA vulvar rejuvenation” protocol in women with mild to severe vulvar atrophy (four 10-min sessions, spaced 14 to 16 d apart, with a 42°C target temperature in vulvar tissues during procedure; range, 40°C to 43°C).^51^ A meta-analysis on four case series for a total of 127 patients revealed that DQRF treatment significantly improved vulvovaginal aesthetic appearance, sexual satisfaction, and symptoms of vulvovaginal atrophy (VVA). On the other hand, the authors critically underlined conflicts of interest as a possible cause of bias of the review, as the included studies had been conducted by members of the Novavision Group. They also stressed that the studies were too small, lacking a control group, and patient selection methods were often not mentioned. DQRF is therefore a potentially promising treatment, although, to definitely assess its efficacy, better designed trials are strongly needed.^29^

To our knowledge, this is the first study to compare the efficacy of DQRF with that of vaginally administered estriol.^24,29,30,32,33,49,53^ The results we obtained show that RF and local estrogens obtain comparable results in terms of patient satisfaction and improvement of GSM symptoms. Currently, there are no randomized controlled trials in the literature comparing the efficacy of DQRF for the treatment of genitourinary syndrome, either against estrogen or against placebo. For the introduction of a new therapy into the market, it is essential to test its efficacy with these types of studies that can provide quality evidence. Therefore, we hope to see such studies in the future.

However, it is necessary to emphasize the difference between the two treatments in terms of costs, both for the patients and for healthcare system. In our protocol, RF treatment involved four sessions, each of 30 min, as the treatment itself lasted 15 min, provided by a healthcare worker (usually either a gynecologist or a midwife).

The total costs of radiofrequency treatment therefore include the cost of the device (in our case, estimated at €28,000 at the time of the study for EVA by Novavision Group SpA), maintenance costs, the cost of the health professional's time, costs related to the treatment site, and also indirect costs related to the time the woman has to invest in the treatment (possible reduction in working hours, transport, etc).

On the other hand, local estrogen represents a low-cost therapy. In Italy, a 20-application packet costs about €7.50, and it is administered at home by the woman herself, with no additional costs. Furthermore, although the follow-up in our study was only 6 mo and although it is unclear in the current state of the literature how long the benefits of radiofrequency devices last, radiofrequency treatments would appear not to be long lasting and would require additional follow-up sessions, so the cost difference would be even more marked.

Therefore, although ET remains the gold standard for its low costs and ease of use, our results are promising and suggest that RF can be considered as an alternative to local hormone therapies in specific cases. We have already underlined how in the last decades there has been an increasing interest in the development of medical devices to relieve GSM symptoms and improve sexual function. This field of interest acquires particular importance for that population of women for whom hormone treatment is not recommended, not tolerated, or not accepted.

There is a great need for safe and efficacious options in GSM treatment for women for whom estrogens are not recommended, such as breast cancer survivors. According to the American College of Obstetricians and Gynecologists (ACOG), first-line approaches to manage GSM in breast cancer survivors are nonhormone options, but when refractory or severe symptoms occur, the use of vaginal estradiol or DHEA can be considered a safe option. Despite this, physicians are often reticent to prescribe estrogens to these patients, and on the other hand, breast cancer survivors may be hesitant to use hormones. For this patient population, radiofrequency treatment could represent a valid option and an additional resource that can be offered to these women for a better quality of life.

### Strengths of the study

Statistical methods based on propensity score matching are increasingly being used to estimate the effects of treatments when randomized clinical trials were not feasible for ethical or practical reasons, and there are only observational data available. The use of propensity score matching is certainly the strength of our study, reducing but not eliminating potential sources of bias and facilitating comparability between the two groups.

Another strength of this study is that it is the first research comparing the efficacy of quadripolar electromagnetic waves versus vaginally administered estriol. Although energy-based treatments represent a new therapeutic option for genital disorders, their efficacy is controversial, and a strong body of evidence is lacking.

### Limitations

The methodology of the research could be considered a limitation, as the study is not a randomized controlled trial. A study based on patient preference introduces an important selection bias, thus limiting the interpretation of the observed results, as comparisons between preference arms have the potential limitations of observational studies. The main difference between an observational study and a patient preference study is the way the treatment is assigned by the researchers in the first case and by the patient's choice in the second. However, it has been recognized that such a research environment may be more similar to “real-world” conditions.^54^

We opted for a patient preference trial as we believe that the active participation of patients in the decision-making process plays a major role in adherence to treatment.^30^ In addition, patients' preferences and lifestyle must be considered when choosing different therapeutic approaches, as selection bias would increase if patients were dissatisfied with their treatment. This is particularly true for patient outcome measures that are subjectively determined, such as patient satisfaction, acceptability, and impact on quality of life. These outcomes are the result of several factors, including tolerance and convenience, which contribute significantly to long-term compliance. In this context, a patient preference study seemed methodologically acceptable. Assignment by the patients themselves does not prevent the potential for bias but may offer some reassurance that results can be extrapolated to a larger group of people, when women have such strong treatment preferences that they refuse randomization. However, in these cases, the effect can be referred exclusively to patients who specifically choose that treatment.^55^

In addition, we must also consider that a patient preference design may create a bias as the final satisfaction may be influenced by initial expectations, as well as by the fact that women involved in the medical decision-making process are likely to have better results than those on whom treatments are imposed.

## CONCLUSIONS

Our study shows that RF is as effective as topical hormone treatment and could be suggested as an alternative to it, selectively in cases in which estrogens are contraindicated, not tolerated, or not effective. Treatment management should be tailored to individual goals and to patient's symptoms. From this perspective, further studies addressing the cost-effectiveness, in addition to the clinical effectiveness, of the two treatment options are needed.
